# Directional Colony Growth of *Cupriavidus* toward Sphingomonads

**DOI:** 10.1264/jsme2.ME25087

**Published:** 2026-04-29

**Authors:** Hiromi Kato, Shoko Hirano, Chiaki Haga, Sayaka Sakogawa, Yoshiyuki Ohtsubo, Yuji Nagata

**Affiliations:** 1 Graduate School of Life Sciences, Tohoku University, 2–1–1 Katahira, Sendai 980–8577, Japan

**Keywords:** *Cupriavidus*, sphingomonads, sphingosine, directional colony growth, interspecies interaction

## Abstract

Bacterial communities often originate from physical encounters between distinct species on solid surfaces; however, the mechanisms underlying these initial interactions remain unclear. The γ-hexachlorocyclohexane (γ-HCH; a toxic recalcitrant insecticide)-degrading consortium includes the surface-motile bacterium *Cupriavidus* sp. strain TKC and the γ-HCH-degrading *Sphingobium* sp. strain TKS. During a co-culture on R2A agar, TKC colonies exhibited directional colony growth (DCG), characterized by asymmetric expansion toward neighboring TKS colonies. When TKC colonies eventually overgrew TKS colonies, non-motile TKS cells were passively carried along the expanding TKC front, indicating that DCG facilitates the physical association and surface dispersal of *Sphingobium* cells. DCG activity was also observed against various strains of *Sphingobium*, *Sphingomonas*, and *Novosphingobium*, whereas extremely weak or no DCG activity was observed toward other bacterial groups, including representatives of α-, β-, and γ-proteobacteria as well as actinobacteria. These results indicate that TKC exhibits DCG selectively toward sphingomonads, recognizing taxon-specific cues rather than responding indiscriminately to neighboring colonies. Among the components tested, sphingosine reproducibly triggered DCG in a dose-dependent manner, and the inhibition of sphingolipid biosynthesis in *Sphingobium* with myriocin markedly suppressed the inducing effect. These results highlight the sphingosine-mediated induction of DCG as a molecular mechanism underlying the initial step in the spatial organization of bacterial communities on solid surfaces.

Bacterial colonies have long been a central focus of microbiology as the foundation of pure culture isolation. Beyond this classical role, their morphogenesis and self-organized branching patterns have also attracted considerable mathematical interest (Matsushita and Fujikawa, 1990; Benjacob *et al.*, 1994; Wakita *et al.*, 1997). In recent years, colonies have further come to be recognized not simply as clonal populations, but as spatially organized, multispecies units in which physical arrangements and local metabolic interactions shape community structures and functions (Estrela and Brown, 2013; Nadell *et al.*, 2016; Borer *et al.*, 2020). This reflects bacteria frequently forming microcolonies and biofilms at solid–liquid interfaces in natural environments, which give rise to cooperative and symbiotic networks (Flemming *et al.*, 2016). However, the very initial “encounter” between heterologous bacterial colonies on solid surfaces has not yet been exami­ned in detail. The mechanisms by which different bacterial species first recognize one another and begin to interact in ways that enable subsequent co-existence remain unclear.

The γ-isomer of hexachlorocyclohexane (γ-HCH) is a persistent organochlorine insecticide that has caused serious environmental contamination (Phillips *et al.*, 2005; Vijgen *et al.*, 2011). Microbial consortia enriched on γ-HCH are typically dominated by sphingomonads, a group of *Alphaproteobacteria* known for their ability to degrade HCH and other xenobiotics (Nagata *et al.*, 2019). Alongside these degraders, non-degrading bacteria are often retained as stable members, suggesting that ecological interactions other than substrate utilization contribute to community compositions (Ogawa *et al.*, 2019; Liu *et al.*, 2025). We previously isolated *Cupriavidus* sp. strain TKC (Kato *et al.*, 2023), which does not degrade γ-HCH, but remains one of the dominant members of the community, from a γ-HCH enrichment culture, together with *Sphingobium* sp. strain TKS, a γ-HCH degrader (Tabata *et al.*, 2016).

In the present study, we found that when co-cultured on agar, TKC colonies exhibited spatially asymmetric expansion toward TKS. We hereafter refer to this colony-level growth behavior—characterized by asymmetric expansion toward a specific external target—as directional colony growth (DCG). This observation suggests the presence of interspecies cues that guide colony expansion and potentially affect the initial assembly of microbial consortia. We further show that DCG is selectively induced by sphingomonads, independent of their γ-HCH-degrading ability, and that lipid components, particularly sphingosine, are likely inducers. These results provide novel insights into the potential molecular mechanisms underlying the early stages of bacterial consortium formation.

## Materials and Methods

### Strains and culture conditions

The bacterial strains used in this study are listed in Table 1. Two surface-motile *Cupriavidus* strains were employed: *Cupriavidus* sp. strain TKC and *C. metallidurans* CH34, a well-characterized representative strain of the genus, for comparison. As DCG target‍ ‍strains representing sphingomonads, we used *Sphingobium* sp. TKS, *S. japonicum* UT26, *Sphingobium* sp. MI1205, *S. chlorophenolicum* L-1, *Sphingobium* sp. SYK-6, *Sphingomonas* sp.‍ ‍MM-1, *S. sanguinis* IAM12578, and *Novosphingobium aromaticivorans* F199. These strains are known degraders of γ-HCH, pentachlorophenol, lignin-derived aromatics, or other aromatic hydrocarbons. In addition, non-sphingomonad bacterial strains, which included *Escherichia coli* BW25113 (a laboratory reference strain), *Sinorhizobium meliloti* 1021 (a root nodule symbiont), *Burkholderia multivorans* ATCC 17616 (an environmental isolate with broad metabolic versatility), and *Rhodococcus jostii* RHA1 (a model actinobacterium capable of PCB and aromatic hydrocarbon degradation), were exami­ned as DCG targets to assess the specificity of the phenomenon. *Cupriavidus* strains were cultivated on R2A agar, whereas all other strains were pre-cultured on one-third-strength LB (1/3 LB) agar plates. All cultures were incubated at 30°C for 3 days in the dark prior to their use in DCG assays.

### Observation of DCG

The precultured cells of TKC and each DCG-target strain were washed with PBS and resuspended in PBS to an OD_660_ of 1.0. Serial dilutions (10^3^ to 10^7^) were prepared for both TKC and each target strain, and equal volumes of the corresponding dilutions were mixed in all pairwise combinations. A 100-μL aliquot of each mixture was spread onto R2A agar plates, which were then incubated at 30°C in the dark. This procedure generated plates in which colonies of the two species were distributed across a wide range of abundances and ratios. In the DCG assay, R2A agar plates were freshly prepared 1 day before the experiment to ensure consistent agar surface conditions across experiments.

Colony morphology was monitored over time using a stereomicroscope equipped with oblique illumination (Nikon SMZ1270). DCG activity was quantified by evaluating the departure from circularity in TKC colonies that grew directionally toward target colonies. In each TKC colony, the length of the major axis was divided by that of the perpendicular minor axis to obtain a deformation ratio. Plates across the dilution series were exami­ned, and DCG activity was judged to occur when at least one TKC colony exhibited a deformation ratio ≥1.1.

### Lipid extraction and DCG assay using lipid fractions

Lipid extraction was performed using a chloroform–methanol (2:1, [v/v]) solvent system, which is commonly applied for the extraction of sphingolipids from sphingomonads (Kawahara *et al.*, 2000) with modifications. *Sphingobium* sp. strain TKS was cultured in 20 mL of 1/3 LB liquid medium, and cells were harvested by centrifugation and washed with PBS. The cell pellet was then‍ ‍resuspended in 2 mL of chloroform–methanol (2:1, [v/v]) by pipetting. Cells were disrupted by sonication (SONIFIER 250; BRANSON) at an intensity of 3 for 30 s. The suspension was centrifuged at 2,500×*g* for 5 min, and the organic phase was collected. The extract was dried under a vacuum (high temperature, 20 min), and the dried material was dissolved in 50 μL of dimethyl sulfoxide (DMSO). This crude organic-soluble extract was hereafter referred to as the lipid fraction, which was used immediately after preparation. In parallel, a cell suspension of *Cupriavidus* sp. strain TKC was prepared and serially diluted to 10^6^–10^7^. Aliquots (100 μL) of each dilution were spread on R2A agar plates, which were incubated at 30°C in the dark. After 3 days of incubation, when TKC colonies had formed, 5 μL of the lipid fraction was spotted onto the agar surface at a distance of 10 mm from the colony edge. As a comparison, a lipid fraction that had been autoclaved and then dissolved in DMSO was also spotted in the same manner (5 μL). The plates were further incubated at 30°C in the dark and were observed under the stereomicroscope. This assay was performed in triplicate. Since the lipid fraction from 20-mL cultures was insufficient for reliable gravimetric quantification, a scale-up extraction from 80-mL cultures was performed under identical conditions. This yielded 8.5 mg of dry material, corresponding to approximately 0.2 mg (dry weight) in the 5-μL aliquot used for the DCG assay.

### Sphingosine and related compound assays

*Cupriavidus* sp. strain TKC was pre-cultured on R2A agar plates at 30°C for 2 days, and *Sphingobium* sp. strain TKS was pre-cultured on 1/3 LB agar plates at 30°C for 4 days. A colony of strain TKC was inoculated onto the plate, and strain TKS or a chemical compound was spotted 10 mm from the TKC inoculation site. The tested compounds were D-sphingosine (>96.0%; Tokyo Chemical Industry) (10, 1.0, 0.1, and 0.01 mg mL^–1^) and α-galactosylceramide (α-GalCer) (>95.0%; Tokyo Chemical Industry) (28 and 2.8 mg mL^–1^). Autoclaved wooden toothpicks were used for inoculation. The compounds were dissolved in DMSO, and 2 μL of each solution was applied to the agar surface. The plates were further incubated at 30°C in the dark and were observed using the stereomicroscope. To inhibit sphingosine biosynthesis, myriocin (An *et al.*, 2011) was added to both 1/3 LB agar (for the pre-culture) and R2A agar (for the DCG assay) at a final concentration of 2 μM. The number of biological replicates per condition ranged from 8 to 15, depending on the treatment (details are described in Fig. 2).

### RNA-seq anal­ysis of TKC during DCG

*Sphingobium* sp. TKS and *Cupriavidus* sp. TKC were used for the RNA-seq anal­ysis. TKS colonies grown on 1/3 LB agar and TKC colonies grown on R2A agar were pre-cultured at 30°C in the dark. Colonies from each strain were then collected and inoculated onto R2A agar plates in pairs, positioned 15 mm apart, with four pairs prepared per plate. As a control, TKC was also cultured alone on R2A agar. After 15 days of incubation at 30°C in the dark, TKC cells were harvested either from the DCG region (the thin, elongated zone extending toward TKS) or from TKC-only colonies. Since the DCG region contained only a very thin cell layer, TKC cells were collected from 56 TKC–TKS pairs and pooled to obtain a sufficient biomass. Under the TKC-only condition, cells were collected from four independent TKC colonies and pooled. Therefore, one pooled RNA sample per condition (DCG and TKC-only) was used for sequencing. Total RNA was extracted using the RNeasy Mini Kit (QIAGEN) according to the manufacturer’s instructions. RNA sequencing was performed using the DBSEQ platform (150×2). The resulting Fastq files were converted to the DAA format using DIAMOND-AnnoTree (Gautam *et al.*, 2022), and a KEGG functional anal­ysis was conducted with MEGAN (version 6.24.23). Read counts were normalized among samples, and Log2FoldChange values (DCG/TKC) were calculated to compare the DCG condition with the TKC-only condition. RNA-seq data have been deposited in the DDBJ Sequence Read Archive under BioProject accession number PRJDB37954 (DRR792201-DRR792202).

## Results and Discussion

### DCG of *Cupriavidus* sp. TKC toward *Sphingobium* sp. TKS

**Fig. 1.**
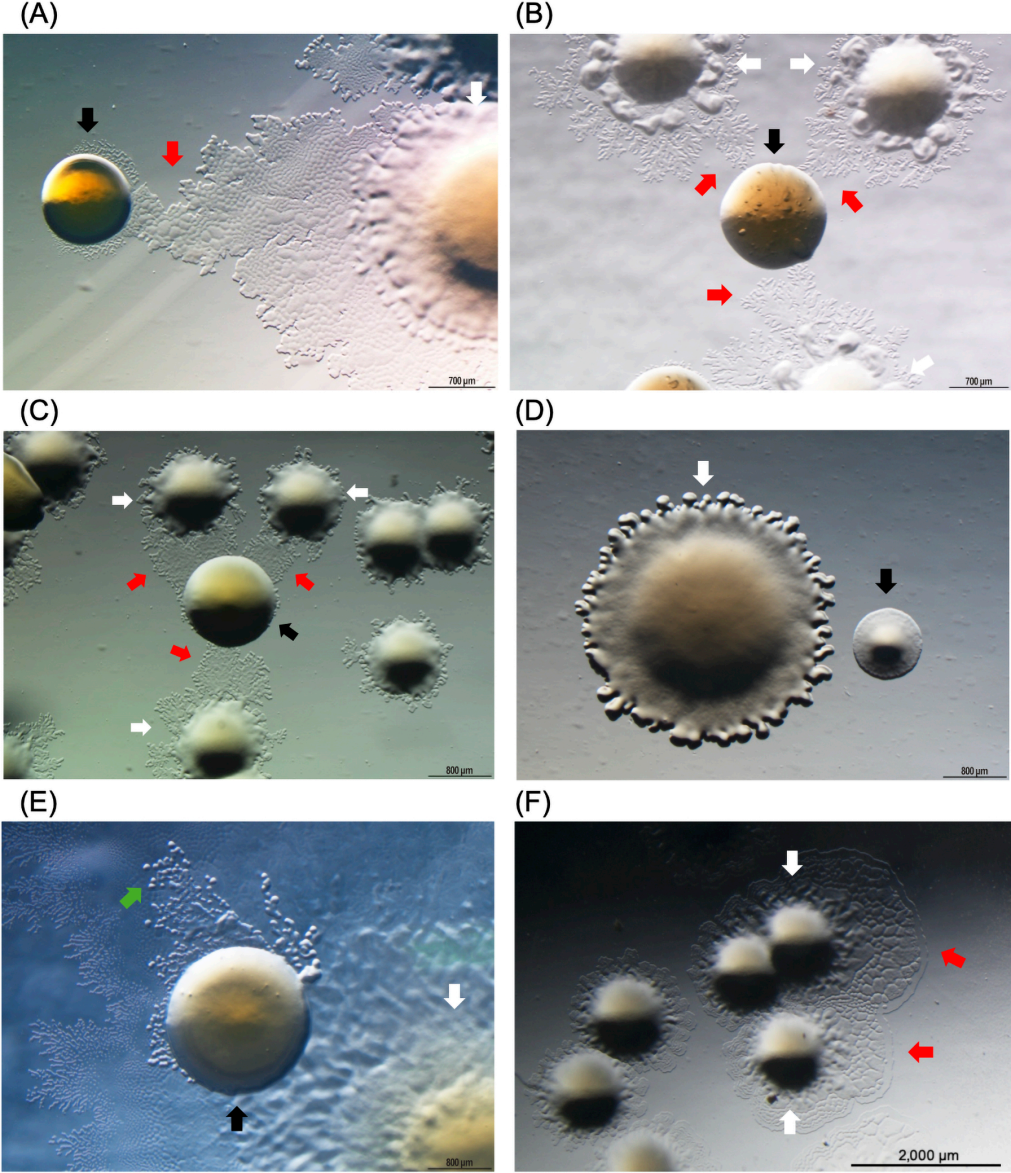
Directional colony growth (DCG) of *Cupriavidus* sp. strain TKC toward *Sphingobium* sp. strain TKS on R2A agar, observed by stereomicroscopy with oblique illumination. (A) Directional elongation of TKC colony margins toward a neighboring *Sphingobium* sp. strain TKS colony. (B) Coordinated DCG responses of multiple TKC colonies positioned around a single TKS colony, in which all TKC colonies exhibited colony expansion directionally toward the same target colony. (C) DCG of TKC in the presence of *Novosphingobium aromaticivorans* strain F199. (D) DCG of TKC in the presence of the non-sphingomonad *Sinorhizobium meliloti* strain 1021. (E) Overgrowth of a TKS colony by TKC, in which the original non-motile TKS cells are redistributed along the direction of TKC expansion, resulting in the formation of multiple small TKS colonies on the upper-left side of the original TKS colony. (F) DCG-like colony expansion of strain TKC induced by an exogenous synthetic surfactant mixture. The surfactant drop center is located outside the field of view (see Supplementary Fig. S6 for the experimental layout). In all panels, black arrows indicate target colonies, white arrows indicate representative TKC colonies exhibiting clear DCG, and red arrows highlight regions exhibiting DCG. The green arrow indicates areas where TKS cells are redistributed during overgrowth. Scale bars are shown in each panel.

*Cupriavidus* sp. strain TKC formed smooth and circular colonies on nutrient-rich media, such as 1/3 LB agar (Fig. S1A). In contrast, when grown on a low-nutrient medium, such as R2A, TKC colonies displayed thinner and radially extended margins (Fig. S1B). When serial dilutions of mixed suspensions of *Cupriavidus* sp. strain TKC and *Sphingobium* sp. strain TKS were plated on R2A agar, TKC colonies exhibited DCG toward neighboring TKS colonies after approximately one to two weeks (Fig. 1A). This phenomenon did not occur uniformly across all colonies on a plate, it was only observed among those located within close proximity to TKS colonies—typically within 7 mm between colony centers, with the farthest case being detected at 20 mm. When multiple TKC colonies were positioned around a single TKS colony, all responsive colonies expanded toward the same target (Fig. 1B).

In contrast to previously described interspecies surface interactions that involve visible biofilm or colony architectures (Molina-Santiago *et al.*, 2019, 2021; Bru *et al.*, 2023), DCG observed in colonies derived from diluted inocula often appeared as an extremely thin, nearly transparent layer. Oblique illumination, previously applied in stereomicroscopic observations of surface-motile bacterial colonies and their interactions (Burchard and Sorongon, 1998; Kientz *et al.*, 2012), was essential for detecting DCG in this experimental setting.

When TKC colonies eventually overgrew TKS colonies, non-motile TKS cells were passively carried along the expanding TKC front, indicating that DCG facilitates the physical association and surface dispersal of *Sphingobium* cells, as supported by PCR-based detection using sphingomonad-specific primers (Leys *et al.*, 2004) at the overgrown areas (Fig. 1E, Supplementary Fig. S2). Similar cargo transport or co-swarming behaviors have been documented in other bacterial systems, where motile populations carry non-motile partners to promote joint expansion or community dispersal (Finkelshtein *et al.*, 2015; Shrivastava *et al.*, 2018). However, these reported phenomena represent interactions that occur after the initial encounter between species, and the mechanisms by which these cooperative associations are first initiated remain unclear. Therefore, the DCG phenomenon described herein may provide a model for the earliest stage of physical engagement between distinct bacterial species.

#### Transcriptomic anal­ysis of TKC during DCG

This colony-level response is distinct from classical chemotaxis, in which individual cells actively swim toward chemical gradients. DCG represents a coordinated expansion of the colony, a growth-associated phenomenon rather than motility driven solely by flagellar swimming. Therefore, DCG shares certain features with swarming, sliding, or gliding motility; however, its precise mechanisms remain unclear. To investigate whether known motility systems contribute to the DCG phenomenon, we conducted a preliminary RNA-seq anal­ysis of cells collected from the advancing front of TKC colonies. However, a marked increase (>four-fold) in the expression of flagellar-, pili-, or secretion-associated motility genes was not detected (Supplementary Table S1 and S2). Previous transcriptomic anal­yses of surface-motile bacteria showed that the expression of motility-related genes at the colony edge was highly dependent on the condition or phase and may markedly vary across studies (Overhage *et al.*, 2008; Tremblay and Déziel, 2010; Pearson *et al.*, 2010; Jeckel *et al.*, 2023). Therefore, the absence of such signatures in our dataset does not necessarily exclude the involvement of known motility systems in DCG. Since our RNA-seq anal­ysis captured only a single time-point snapshot of transcriptional activity, it may have missed transient expression changes that occurred before or after the sampling time.

#### Taxonomic specificity of DCG

To evaluate the taxonomic breadth of this phenomenon, the DCG activity of strain TKC was exami­ned against various bacterial strains (Table 1, Fig. 1C, 1D, S3, and S4). This activity was quantified as the major-to-minor axis ratio of the colony. Against *Sphingobium japonicum* UT26, a model strain for γ-HCH degradation, TKC displayed a DCG index of approximately 1.3, which was similar to that observed with TKS. Equivalent responses were also observed toward other sphingomonads, including members of the genera *Sphingomonas* and *Novosphingobium* (Fig. 1C). Notably, the presence or absence of γ-HCH-degrading ability among these strains did not affect the induction of DCG activity. In contrast, when tested against non-sphingomonad strains, including the alphaproteobacterium *Sinorhizobium meliloti* as well as other *Proteobacteria* and *Actinobacteria*, the DCG index remained close to 1.0, indicating little or no activity (Table 1, Fig. 1D and S4). These results suggest that TKC exhibited DCG activity selectively toward sphingomonads, recognizing a taxonomically restricted group of bacteria.

The taxonomic restriction of DCG activity to sphingomonads indicates that TKC recognizes lineage-specific cues rather than responding indiscriminately to neighboring colonies. This specificity is notable because sphingomonads encompass diverse metabolic traits, including both γ-HCH-degrading and non-degrading strains; however, TKC responded similarly to both groups. Therefore, the presence of DCG activity cannot be explained simply by shared catabolic functions, but instead points to conserved lineage-associated features.

### Sphingolipid-derived compounds as candidate inducers of DCG

**Fig. 2.**
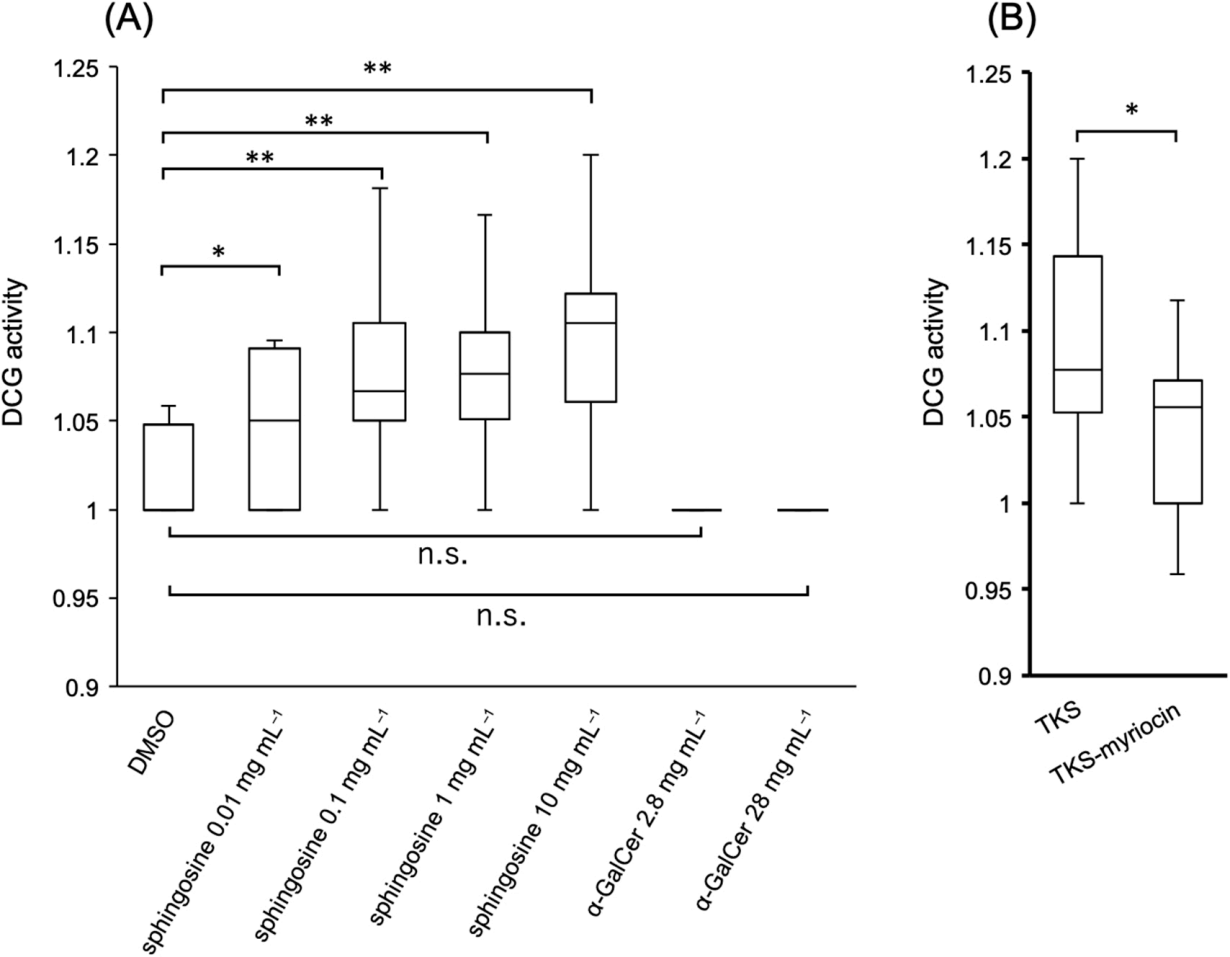
Induction of directional colony growth (DCG) by sphingolipid-related compounds. (A) DCG activity of *Cupriavidus* sp. strain TKC in response to dimethyl sulfoxide (DMSO; control), sphingosine, and α-galactosylceramide (α-GalCer). (B) DCG activity toward *Sphingobium* sp. strain TKS cultured in the presence or absence of myriocin. DCG activity was evaluated as the ratio of the long to short axis of the TKC colony. The significance of differences was assessed using the Wilcoxon rank-sum test (*, *P*<0.05; **, *P*<0.0005; n.s., not significant). The number of biological replicates was as follows: TKS (*n*=15), DMSO (*n*=14), sphingosine 0.01 mg mL^–1^ (*n*=15), sphingosine 0.1 mg mL^–1^ (*n*=15), sphingosine 1 mg mL^–1^ (*n*=13), sphingosine 10 mg mL^–1^ (*n*=10), α-GalCer 2.8 mg mL^–1^ (*n*=13), α-GalCer 28 mg mL^–1^ (*n*=8), and TKS treated with myriocin (*n*=15). Outliers were excluded from the anal­ysis.

The strong taxonomic specificity of DCG toward sphingomonads suggested that TKC responded to lineage-specific chemical cues. A notable lineage-specific trait of sphingomonads is their membrane composition. While the outer membrane of most Gram-negative bacteria contains lipopolysaccharides, sphingomonads possess glycosphingolipids (Sohlenkamp and Geiger, 2016). This unique feature may contribute to the high surface hydrophobicity of sphingomonads and their ability to degrade recalcitrant compounds. Based on this distinctive membrane property, we focused on cellular lipids to investigate whether candidate factors capable of inducing DCG activity may be identified. The lipid fraction of TKS cells was sufficient to induce DCG activity in TKC (DCG index=1.27±0.01, mean±SD). To identify specific lipid components responsible for this activity, we selected sphingosine, a major structural element of these lipids, as a candidate inducer and experimentally evaluated its effects on DCG activity. Although the induction observed with sphingosine was not as strong as that with whole-cell lipid extracts, sphingosine increased DCG activity in TKC significantly more than the DMSO control (Fig. 2A). In addition, increasing concentrations of sphingosine were generally associated with stronger DCG responses. The reduction in DCG induction when TKS cells were cultured in the presence of myriocin further supports a role for sphingolipid biosynthesis in this phenomenon (Fig. 2B). As a comparison, we also tested α-GalCer, a synthetic sphingolipid analog that is not known to occur in sphingomonads. α-GalCer did not induce DCG activity (Fig. 2A), supporting the view that the response is associated with naturally occurring sphingolipid components of sphingomonads. Representative images corresponding to these quantitative anal­yses are shown in Supplementary Fig. S5. Under toothpick-inoculation conditions, DCG appeared as simple colony asymmetry rather than the thin, highly dendritic spreading layer observed for single colonies derived from diluted inocula (Fig. 1).

Sphingolipid extraction from sphingomonads yields complex mixtures, making the selective isolation of individual sphingolipids technically challenging. Therefore, we employed sphingosine as a chemically defined and commercially available sphingolipid-derived component, enabling the reproducible evaluation of its effects on DCG. Using this approach, sphingosine reproducibly induced DCG in TKC, supporting the function of sphingolipid-related molecules as DCG-inducing cues. However, extracellular sphingosine released from TKS was not directly quantified in this study, and the mechanisms by which these molecules are released, diffuse across agar surfaces, and reach neighboring colonies remain unclear. The spatial scale of DCG suggests that extracellular diffusion contributes to signal propagation, potentially facilitated by membrane-derived structures such as outer membrane vesicles released by sphingomonads (De Lise *et al.*, 2019).

In addition to their structural roles in eukaryotic membranes, sphingoid bases, such as sphingosine, may act‍ ‍as signaling molecules in bacteria. In *Pseudomonas aeruginosa*, sphingosine directly binds to the transcriptional regulator SphR, thereby inducing the expression of ceramidase genes (LaBauve and Wargo, 2014; Okino and Ito, 2016). This SphR-mediated system exemplifies a receptor–ligand response cascade in which sphingosine functions as an intracellular signal. More recent work has expanded the SphR regulon, demonstrating the sphingosine-dependent activation of virulence-associated factors including phospholipase C (Mackinder *et al.*, 2024). Although no gene with strong similarity to *sphR* has been identified in the TKC genome, it remains possible that TKC recognizes sphingosine through an alternative sensory system and exhibits an active response leading to DCG. This interpretation is consistent with the behavioral tendencies reported for members of the genus *Cupriavidus*. *C. necator*, the type species of the genus, was historically described as a “non-obligate bacterial predator” in soil (Makkar and Casida, 1987), indicating that *Cupriavidus* was originally proposed in the context of interbacterial interactions. Consistent with this lineage-level tendency, DCG activity was also observed with another well-studied *Cupriavidus* strain, *C. metallidurans* CH34 (Table 1, Fig. S3H). Although further surveys across diverse *Cupriavidus* lineages are needed, the presence of DCG activity in multiple species suggests that members of this genus have evolved mechanisms to actively recognize and move toward specific bacterial partners.

Alternatively, sphingosine may contribute to the induction of DCG through its amphiphilic property. In TKC, the application of an exogenous synthetic surfactant mixture (Tween 20, Tween 80, and Triton X-100) also produced colony expansion patterns (Fig. 1F, Supplementary Fig. S6). TKC colonies within approximately 10 mm of the drop center showed uniform peripheral thickening, whereas colonies beyond this boundary exhibited directional colony expansion toward the drop center. The distance-dependent responses observed upon surfactant application are consistent with a global reduction in surface tension near the application site, resulting in isotropic expansion, whereas directional expansion emerges when such effects are spatially uneven. However, the extent to which DCG induced by sphingosine reflects an active, cell-mediated response of TKC, passive physicochemical effects associated with amphiphilicity, or a combination of both currently remains unclear. The relative contributions of these mechanisms need to be clarified in further studies using experimental systems that systematically control inoculation conditions, cell density, and spatial arrangements. Under different experimental conditions, directional growth may appear as either highly dendritic expansion or simple colony asymmetry, suggesting that the DCG morphology reflects the relative contribution of active cellular responses and passive physicochemical effects.

The present results do not exclude the possibility that additional extracellular factors also participate in the induction of DCG. Sphingomonads have been reported to release diffusible compounds that promote the growth of other bacteria (Tanaka *et al.*, 2005; Bhuiyan *et al.*, 2016); however, the relationship between these factors and DCG-inducing activity remains unclear. Therefore, DCG may be triggered by a combination of sphingolipid-derived signals and other metabolites acting in concert during the early stages of colony interactions.

## Conclusion

The present study identified DCG in *Cupriavidus* sp. strain TKC as a novel colony-level response selectively induced by sphingomonads. This activity was not linked to metabolic traits, such as γ-HCH degradation, but instead appeared to rely on lineage-associated cues, with sphingolipid-derived components, including sphingosine, emerging as candidate inducers. Unlike predatory interactions, DCG occasionally coincided with the enhanced expansion of the partner colony, suggesting a cooperative rather than antagonistic outcome. A plausible biological role for DCG is the recruitment of motile partners and the facilitation of co-migration, providing a potential entry point for the formation of mixed-species consortia. Importantly, the present results were obtained on agar surfaces, a model system that represents an interface of solid, liquid, and gas phases. These boundary layers are also abundant in natural environments, such as soil, where micro-scale interfaces shape microbial assembly. Therefore, DCG may reflect an ecologically relevant mechanism that contributes to the earliest stages of community establishment at environmental interfaces.

## Citation

Kato, H., Hirano, S., Haga, C., Sakogawa, S., Ohtsubo, Y., and Nagata, Y. (2026) Directional Colony Growth of *Cupriavidus* toward Sphingomonads. *Microbes Environ ***41**: ME25087.

https://doi.org/10.1264/jsme2.ME25087

## Supplementary Material

Supplementary Material

## Figures and Tables

**Table 1.** Directional colony growth (DCG) of *Cupriavidus* sp. strain TKC toward various bacterial target strains on R2A agar

DCG target strain	DCG index	DCG activity	Notable feature	Reference
*Sphingobium* sp. TKS	1.3	+	HCH degrader	Tabata *et al.*, 2016
*Sphingobium* sp. TKS (CH34)*	1.2	+		
*Sphingobium japonicum* UT26	1.3	+	HCH degrader	Imai *et al.*, 1991: Nagata *et al.*, 2011
*Sphingobium* sp. MI1205	1.2	+	HCH degrader	Ito *et al.*, 2007
*Sphingobium chlorophenolicum* L-1	1.4	+	Pentachlorophenol degrader	Copley *et al.*, 2012
*Sphingobium* sp. SYK-6	1.3	+	Lignin-derived aromatic degrader	Masai *et al.*, 2007
*Sphingomonas* sp. MM-1	2.1	+	HCH degrader	Tabata *et al.*, 2011
*Sphingomonas sanguinis* IAM12578	1.4	+	Polyethylene glycol degrader	Takeuchi *et al.*, 1993
*Novosphingobium aromaticivorans* F199	1.1	+	Aromatic hydrocarbon degrader	Fredrickson *et al.*, 1991
*Escherichia coli* BW25113	1.0	–	Enteric bacterium	Datsenko and Wanner, 2000
*Sinorhizobium meliloti* 1021	1.0	–	Root nodule symbiont	Meade *et al.*, 1982; Galibert *et al.*, 2001
*Burkholderia multivorans* ATCC17616	0.9	–	Environmental isolate with broad metabolic versatility	Stanier *et al.*, 1966; Nishiyama *et al.*, 2010
*Rhodococcus jostii* RHA1	1.0	–	PCB and aromatic hydrocarbon degrader	Seto *et al.*, 1995

* DCG was observed for *Cupriavidus metallidurans* strain CH34 instead of *Cupriavidus* sp. strain TKC.
