# Supplementary material for: Directional Colony Growth of Cupriavidus toward Sphingomonads
Source: Microbes Environ. 2026 Apr 29;41(2):ME25087. doi: 10.1264/jsme2.ME25087 (PMC13293705; doi:10.1264/jsme2.ME25087)
Supplement: Supplementary file 1 — Supplementary Material [file 41_25087_s1.pdf]

Directional colony growth of *Cupriavidus* towards sphingomonads

Hiromi Kato*[1], Shoko Hirano*[1], Chiaki Haga, [1] Sayaka Sakogawa[1], Yoshiyuki Ohtsubo[1], Yuji Nagata[1]

Supplementary Materials

Supplementary Table S1-S2

Supplementary Figures S1-S6

Supplementary Table S1. RNA-seq-identified genes showing ≥4-fold differential expression in TKC during directional colony growth.

Annotation based on KEGG ortholog	TKC_DCG	TKC_only	DCG/TKC_only
K18053	295	2	147.6
K1000196 Photosynthesis - antenna proteins	98	1	98.0
K19691	43	1	42.6
K14439	32	2	15.8
K03366 meso-butanediol dehydrogenase / (S,S)-butanediol dehydrogenase / diacetyl reductase [EC:1.1.1.- 1.1.1.76 1.1.1.304]	15	1	14.9
K13688	66	5	13.3
K00847 fructokinase [EC:2.7.1.4]	13	1	12.9
K20170	55	5	11.1
K07319	11	1	10.9
K18132	20	2	9.9
K11418	28	3	9.2
K17832	9	1	8.9
K00683	8	1	7.9
K01278	7	1	6.9
K18360	7	1	6.9
K10715 two-component system, sensor histidine kinase RpfC [EC:2.7.13.3]	40	6	6.6
K02155 V-type H+-transporting ATPase 16kDa proteolipid subunit	13	2	6.4
K01684 galactonate dehydratase [EC:4.2.1.6]	6	1	5.9
K01082 3'(2'), 5'-bisphosphate nucleotidase [EC:3.1.3.7]	6	1	5.9
K01478 arginine deiminase [EC:3.5.3.6]	6	1	5.9
K09883 cobaltochelatase CobT [EC:6.6.1.2]	6	1	5.9
K03199 type IV secretion system protein VirB4	6	1	5.9
K03727	6	1	5.9
K06880	6	1	5.9
K13013	6	1	5.9
K00479	12	2	5.9
K21430	16	3	5.3
K01739 cystathionine gamma-synthase [EC:2.5.1.48]	15	3	5.0
K00813 aspartate aminotransferase [EC:2.6.1.1]	5	1	5.0
K00260 glutamate dehydrogenase [EC:1.4.1.2]	10	2	5.0
K10717 cytokinin trans-hydroxylase	10	2	5.0
K19641	10	2	5.0
K04080	32	7	4.5
K21746	27	6	4.5
K01461	64	15	4.3
K03891 ubiquinol-cytochrome c reductase cytochrome b subunit	4	1	4.0
K00635 diacylglycerol O-acyltransferase [EC:2.3.1.20]	4	1	4.0
K13786 cob(II)yrinic acid a,c-diamide reductase [EC:1.16.8.1]	4	1	4.0
K04100 protocatechuate 4,5-dioxygenase, alpha chain [EC:1.13.11.8]	8	2	4.0
K11252 histone H2B	8	2	4.0
K01174	4	1	4.0
K01302	4	1	4.0
K01449	8	2	4.0
K02853	4	1	4.0
K03709	8	2	4.0
K03932	4	1	4.0
K08219	8	2	4.0
K14272	4	1	4.0
K17675	4	1	4.0
K18398	4	1	4.0
K21181	8	2	4.0
K12542	12	3	4.0

The complete gene-level RNA-seq dataset is available from the DDBJ Sequence Read Archive (BioProject PRJDB37954).

Supplementary Table S2. RNA-seq analysis of motility- and surface-associated genes examined in this study.

Annotation based on KEGG ortholog	TKC_DCG	TKC_only	DCG/TKC_only
**Flagellar**			
K02402 flagellar transcriptional activator FlhC	1797	2699	0.7
K02403 flagellar transcriptional activator FlhD	8426	14204	0.6
K02405 RNA polymerase sigma factor for flagellar operon FliA	710	554	1.3
K02406 flagellin	5285	4280	1.2
K02410 flagellar motor switch protein FliG	1011	758	1.3
K02416 flagellar motor switch protein FliM	1169	1091	1.1
K02417 flagellar motor switch protein FliN/FliY	677	562	1.2
K1002040 Flagellar assembly	58088	60320	1.0
K02386 flagella basal body P-ring formation protein FlgA	1534	1429	1.1
K02387 flagellar basal-body rod protein FlgB	1027	1041	1.0
K02388 flagellar basal-body rod protein FlgC	678	661	1.0
K02389 flagellar basal-body rod modification protein FlgD	1154	1248	0.9
K02390 flagellar hook protein FlgE	1992	2129	0.9
K02391 flagellar basal-body rod protein FlgF	1016	1050	1.0
K02392 flagellar basal-body rod protein FlgG	1138	1385	0.8
K02393 flagellar L-ring protein precursor FlgH	1045	1247	0.8
K02394 flagellar P-ring protein precursor FlgI	1799	1531	1.2
K02396 flagellar hook-associated protein 1 FlgK	2592	2093	1.2
K02397 flagellar hook-associated protein 3 FlgL	1520	1294	1.2
K02398 negative regulator of flagellin synthesis FlgM	589	647	0.9
K02399 flagella synthesis protein FlgN	811	865	0.9
K02400 flagellar biosynthesis protein FlhA	2831	2457	1.2
K02401 flagellar biosynthetic protein FlhB	1690	1497	1.1
K02407 flagellar hook-associated protein 2	4007	3395	1.2
K02408 flagellar hook-basal body complex protein FliE	475	321	1.5
K02409 flagellar M-ring protein FliF	2338	2213	1.1
K02411 flagellar assembly protein FliH	1355	1393	1.0
K02412 flagellum-specific ATP synthase [EC:3.6.3.14]	2124	1599	1.3
K02413 flagellar FliJ protein	721	611	1.2
K02414 flagellar hook-length control protein FliK	848	821	1.0
K02418 flagellar protein FliO/FliZ	1101	904	1.2
K02419 flagellar biosynthetic protein FliP	918	779	1.2
K02420 flagellar biosynthetic protein FliQ	243	285	0.9
K02421 flagellar biosynthetic protein FliR	959	740	1.3
K02422 flagellar protein FliS	558	538	1.0
K02423 flagellar protein FliT	419	323	1.3
K13593 cyclic-di-GMP phosphodiesterase, flagellum assembly factor TipF	0	3	na
K10941 sigma-54 specific transcriptional regulator, flagellar regulatory protein A	2086	1551	1.3
**Chemotaxis**			
K00575 chemotaxis protein methyltransferase CheR [EC:2.1.1.80]	1264	1382	0.9
K02556 chemotaxis protein MotA	964	1089	0.9
K03406 methyl-accepting chemotaxis protein	16323	14200	1.1
K03407 two-component system, chemotaxis family, sensor kinase CheA [EC:2.7.13.3]	2292	1988	1.2
K03408 purine-binding chemotaxis protein CheW	995	780	1.3
K03412 two-component system, chemotaxis family, response regulator CheB [EC:3.1.1.61]	2715	2672	1.0
K03413 two-component system, chemotaxis family, response regulator CheY	2828	3412	0.8
K03415 two-component system, chemotaxis family, response regulator CheV	1000	786	1.3
K05874 methyl-accepting chemotaxis protein I, serine sensor receptor	7636	6238	1.2
K05875 methyl-accepting chemotaxis protein II, aspartate sensor receptor	1316	1110	1.2
K05876 methyl-accepting chemotaxis protein III, ribose and galactose sensor receptor	25	12	2.1
K05877 methyl-accepting chemotaxis protein IV, peptide sensor receptor	81	115	0.7
K06596 chemosensory pili system protein ChpA (sensor histidine kinase/response regulator)	7687	6095	1.3
K06597 chemosensory pili system protein ChpB (putative protein-glutamate methylesterase)	0	21	na
K11354 two-component system, chemotaxis family, sensor kinase Cph1 [EC:2.7.13.3]	12	5	2.4
K11444 two-component system, chemotaxis family, response regulator WspR	1279	1096	1.2
K13486 chemotaxis protein methyltransferase WspC	1860	1477	1.3
K13487 methyl-accepting chemotaxis protein WspA	2468	2369	1.0
K13488 chemotaxis-related protein WspB	841	1043	0.8
K13489 chemotaxis-related protein WspD	1107	772	1.4
K13490 two-component system, chemotaxis family, sensor histidine kinase and response regulator WspE	2759	2224	1.2
K13491 two-component system, chemotaxis family, response regulator WspF [EC:3.1.1.61]	11	17	0.6
K13924 two-component system, chemotaxis family, CheB/CheR fusion protein [EC:2.1.1.80 3.1.1.61]	56	33	1.7
K1002030 Bacterial chemotaxis	45394	40508	1.1
K02557 chemotaxis protein MotB	1278	1141	1.1
K03410 chemotaxis protein CheC	0	1	na
K03411 chemotaxis protein CheD [EC:3.5.1.44]	745	688	1.1
K03414 chemotaxis protein CheZ	722	567	1.3
K1004062 Chemokine signaling pathway	9	21	0.4

Supplementary Table S2. RNA-seq analysis of motility- and surface-associated genes examined in this study. (continued)

Annotation based on KEGG ortholog	TKC_DCG	TKC_only	DCG/TKC_only
**Pili**			
K02487 type IV pili sensor histidine kinase and response regulator	465	355	1.3
K02650 type IV pilus assembly protein PilA	1813	1813	1.0
K02657 twitching motility two-component system response regulator PilG	1264	1428	0.9
K02658 twitching motility two-component system response regulator PilH	638	724	0.9
K02659 twitching motility protein PilI	868	626	1.4
K02660 twitching motility protein PilJ	3103	2499	1.2
K02667 two-component system, NtrC family, response regulator PilR	4967	3686	1.3
K02668 two-component system, NtrC family, sensor histidine kinase PilS [EC:2.7.13.3]	2853	2279	1.3
K06596 chemosensory pili system protein ChpA (sensor histidine kinase/response regulator)	7687	6095	1.3
K06597 chemosensory pili system protein ChpB (putative protein-glutamate methylesterase)	0	21	na
K02651 pilus assembly protein Flp/PilA	889	860	1.0
K10924 MSHA pilin protein MshA	0	5	na
K10927 MSHA pilin protein MshD	0	9	na
**Secretion system**			
K03194 type IV secretion system protein VirB1	2	10	0.2
K03195 type IV secretion system protein VirB10	10	4	2.5
K03196 type IV secretion system protein VirB11	118	95	1.2
K03197 type IV secretion system protein VirB2	2	0	na
K03198 type IV secretion system protein VirB3	2	0	na
K03199 type IV secretion system protein VirB4	6	1	5.9
K03200 type IV secretion system protein VirB5	3	0	na
K03201 type IV secretion system protein VirB6	4	0	na
K03203 type IV secretion system protein VirB8	6	0	na
K03204 type IV secretion system protein VirB9	1	1	1.0
K03205 type IV secretion system protein VirD4	43	75	0.6
K03222 type III secretion protein J	5	21	0.2
K03226 type III secretion protein R	0	17	na
K11891 type VI secretion system protein ImpL	79	65	1.2
K11892 type VI secretion system protein ImpK	2	0	na
K11903 type VI secretion system secreted protein Hcp	0	4	na
K11904 type VI secretion system secreted protein VgrG	7921	6406	1.2
K11906 type VI secretion system protein VasD	324	274	1.2
K11907 type VI secretion system protein VasG	981	832	1.2
K11913 type VI secretion system protein	0	1	na
K02487 type IV pili sensor histidine kinase and response regulator	465	355	1.3
K02650 type IV pilus assembly protein PilA	1813	1813	1.0

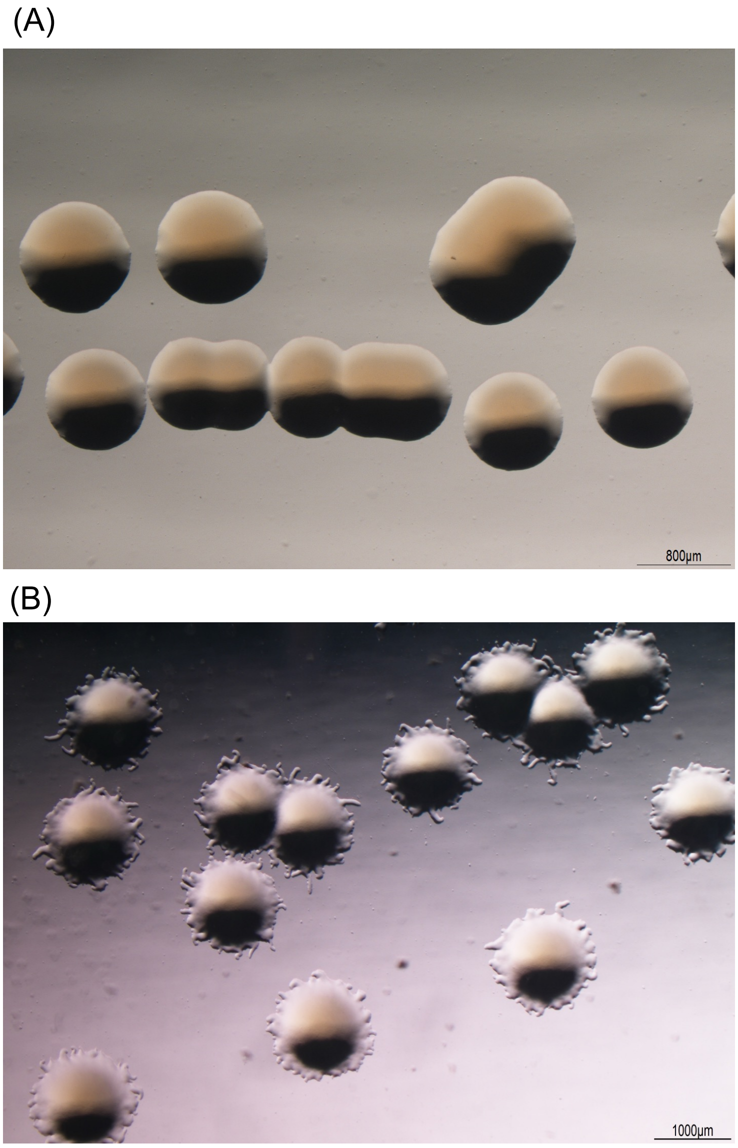

Supplementary Fig. S1. Medium-dependent colony morphology of *Cupriavidus* sp. strain TKC.

(A) On nutrient-rich 1/3 LB agar, TKC formed smooth, circular colonies with well-defined margins. (B) On the relatively low-nutrient R2A agar, colonies exhibited thinner, irregular and radially extended margins, forming a dendritic-like growth pattern. Colonies were as visualized by stereomicroscopy with oblique illumination Scale bars are shown in each panel.

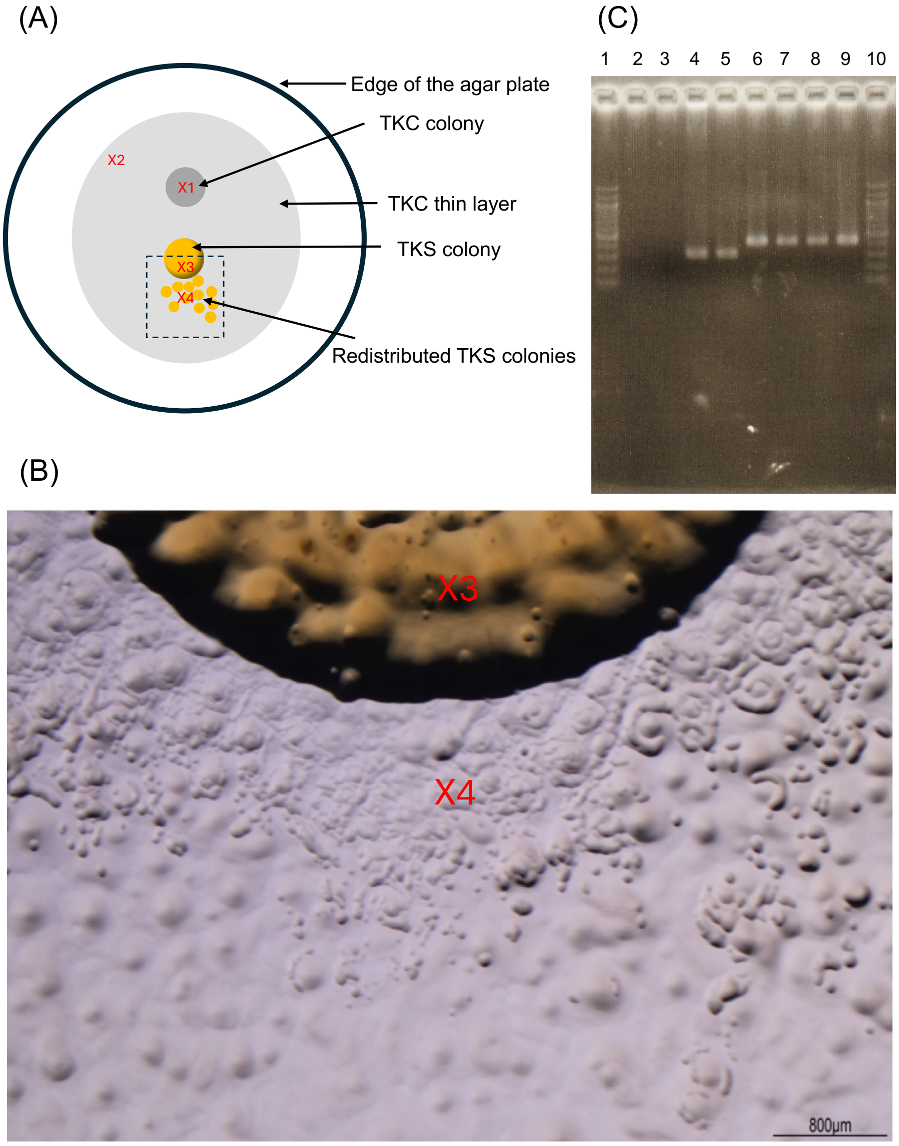

Supplementary Fig. S2. PCR- and culture-based confirmation of Sphingobium cells in TKC overgrowth regions.

(A) Schematic illustration of sampling positions during TKC colony expansion. TKC colonies are shown in gray, the surrounding TKC thin layer in light gray, and the original TKS colony together with redistributed colonies in yellow. X1 indicates the TKC colony, X2 indicates the surrounding TKC thin layer lacking redistributed TKS colonies, X3 indicates the original TKS colony, and X4 indicates redistributed TKS colonies within the overgrowth region. The dashed box corresponds to the microscopic field shown in (B). (B) Representative microscopic image of an overgrowth sample showing the original TKS colony and small colonies redistributed in the direction of TKC expansion. (C) PCR amplification using sphingomonad-specific primers (Leys et al., 2004; lanes 2–5, corresponding to X1–X4) and *Cupriavidus*-specific primers (originally designed for the *Burkholderia*–*Cupriavidus*–*Ralstonia* group; Bauernfeind et al., 1999 *J Clin Microbiol* 37: 1335–1339; lanes 6–9, corresponding to X1–X4). Lane 1 and 10 indicate DNA size markers. Sphingomonad-specific amplification was detected from X3 and X4, but not from X1 or X2. In contrast, *Cupriavidus*-specific amplification was detected from all positions. To exclude the possibility that PCR signals originated from dead cells, redistributed colonies (X4) were re-plated onto fresh R2A agar, resulting in the emergence of both TKC and TKS colonies. Together, these results provide molecular and culture-based evidence that viable TKS cells are physically redistributed within the TKC overgrowth region.

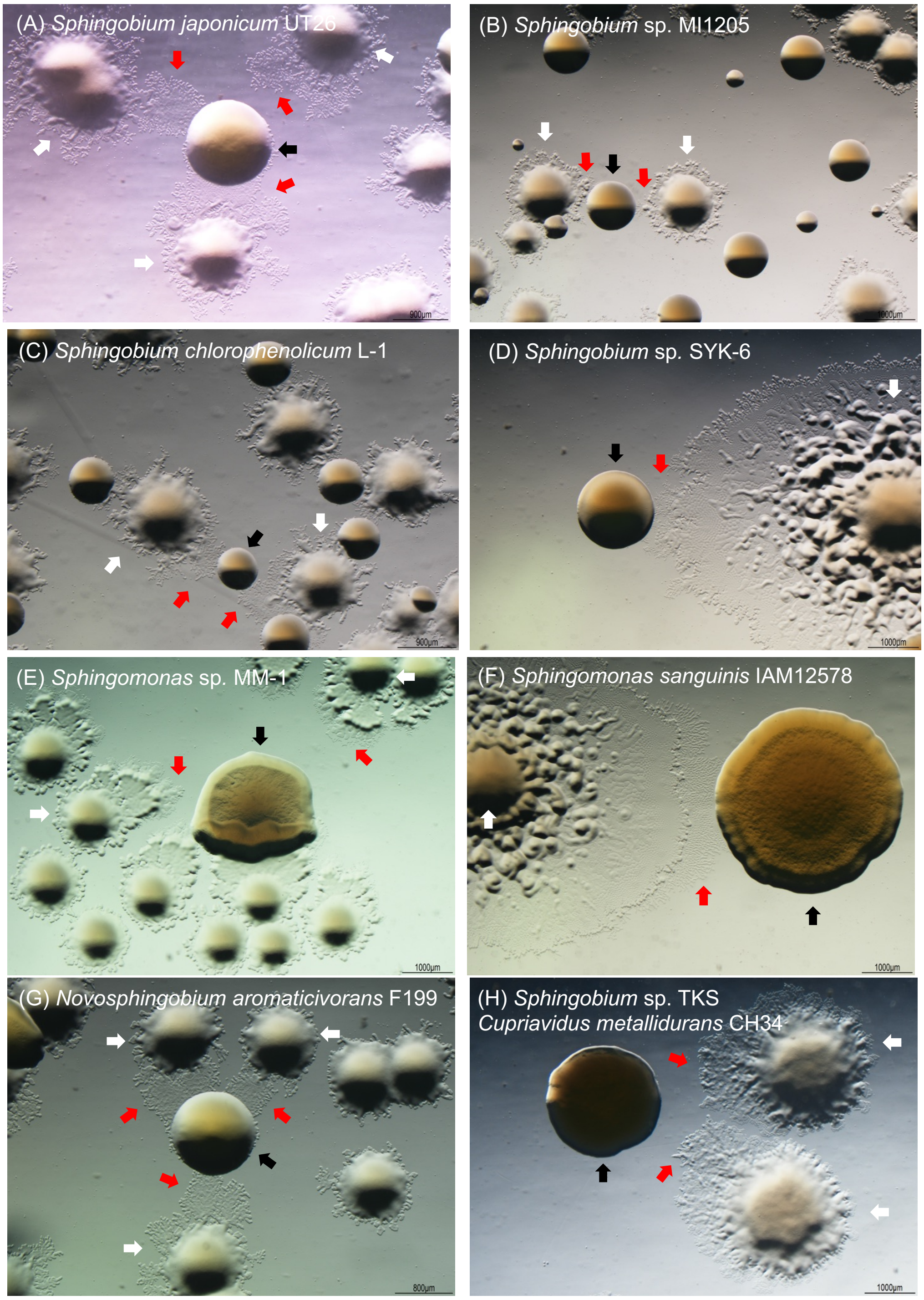

Supplementary Fig. S3. Directional colony growth toward sphingomonads.

Directional colony growth (DCG) of *Cupriavidus* sp. strain TKC was observed in the presence of sphingomonad strains on R2A agar, as visualized by stereomicroscopy with oblique illumination (A-G). The target strains are indicated in each panel. In addition, *Cupriavidus metallidurans* strain CH34 also exhibited DCG activity toward *Sphingobium* sp. strain TKS (H). In all panels, black arrows indicate target colonies, white arrows indicate representative TKC colonies exhibiting clear DCG, and red arrows highlight regions exhibiting directional colony growth. Scale bars are shown in each panel.

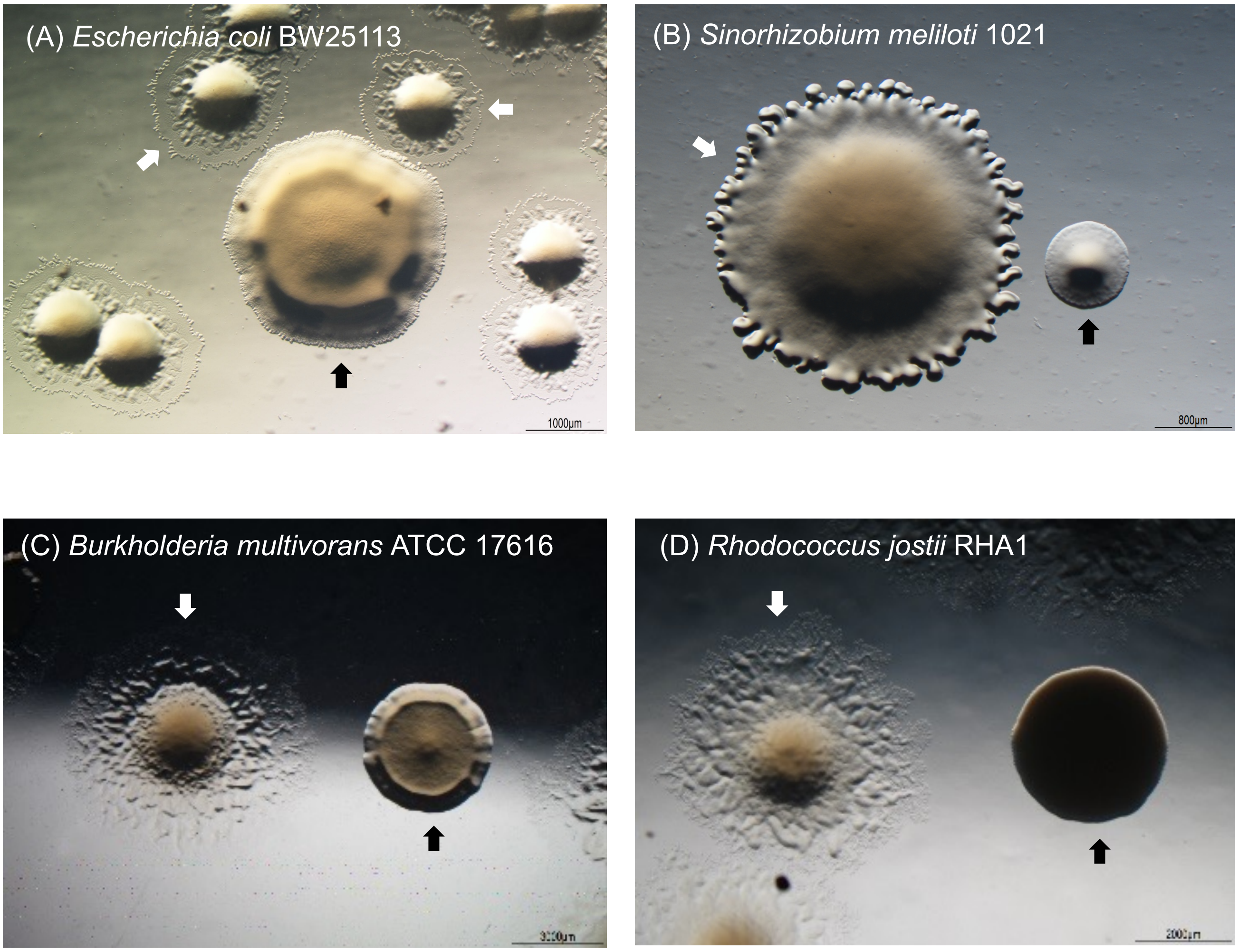

Supplementary Fig. S4. Little or no directional colony growth of *Cupriavidus* sp. strain TKC toward non-sphingomonad strains

Directional colony growth (DCG) of *Cupriavidus* sp. strain TKC was little or no in the presence of non-sphingomonad strains on R2A agar, as visualized by stereomicroscopy with oblique illumination (A–D). The target strains are indicated in each panel. In all panels, black arrows indicate target colonies, white arrows indicate TKC colonies. Scale bars are shown in each panel.

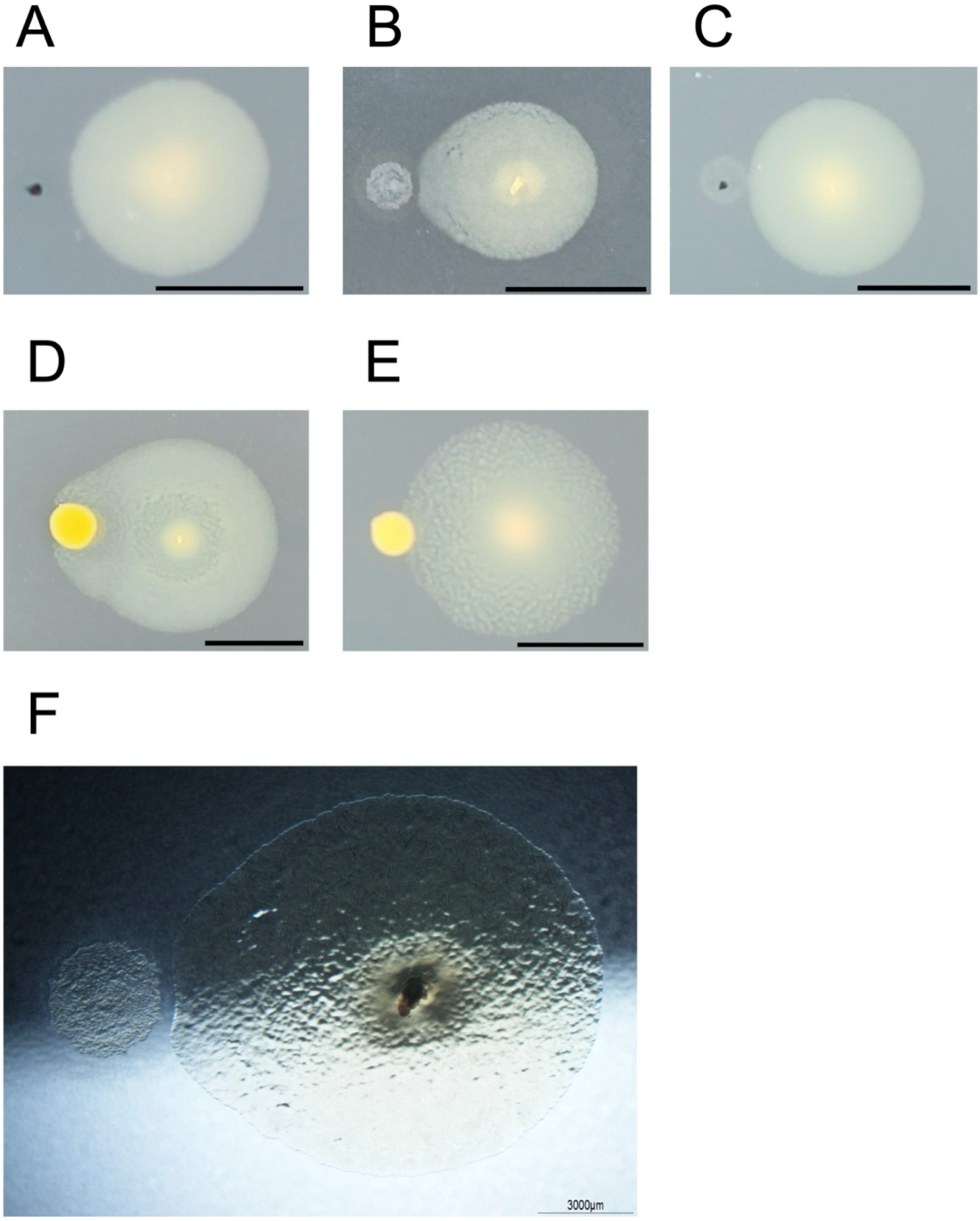

Supplementary Fig. S5. Representative images of directional colony growth (DCG) induced by sphingolipid-related compounds and TKS colonies.

(A) DMSO (control). (B) Sphingosine. (C) α-Galactosylceramide (α-GalCer). (D) *Sphingobium* sp. strain TKS. (E) TKS treated with myriocin. (F) Stereomicroscopic image of the same sphingosine-treated sample shown in (B), obtained with oblique illumination. In panels (A) and (C), the center of the drop is indicated by a black dot because the applied droplet is not clearly visible. Scale bars indicate 10 mm in panels (A–E).

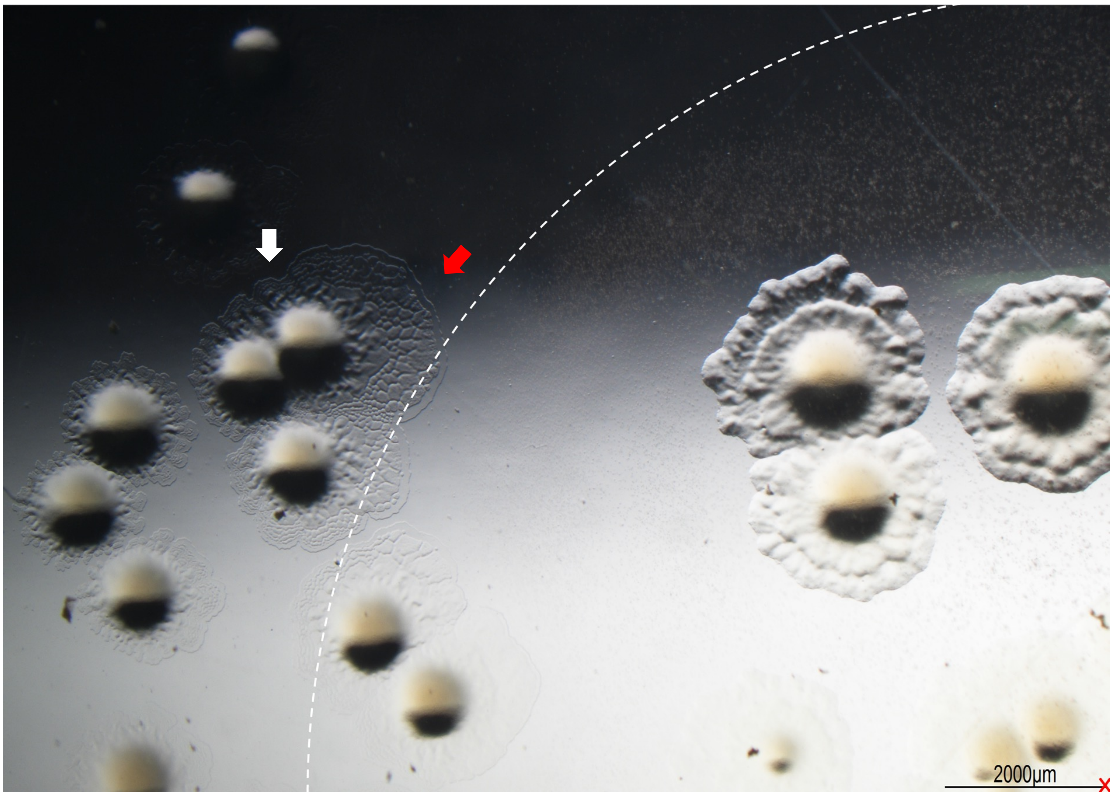

Supplementary Fig. S6. DCG-like colony expansion of *Cupriavidus* sp. strain TKC induced by an exogenous synthetic surfactant mixture, showing the experimental layout.

The center of surfactant application is marked by a red "X", and the boundary corresponding to a 10 mm radius from the drop center is indicated by a white dashed circle. TKC colonies located within this radius showed uniform peripheral thickening, whereas colonies beyond this boundary exhibited directional colony expansion toward the drop center. The region shown in Fig. 1F corresponds to an enlarged area outside the dashed circle, where directional colony expansion is clearly visible. In all panels, a white arrow indicate representative TKC colonies exhibiting DCG-like expansion, and a red arrow highlight region of the expansion. Scale bar is shown.
